# Genome-wide identification and expression profile analysis of SWEET genes in Chinese jujube

**DOI:** 10.7717/peerj.14704

**Published:** 2023-01-17

**Authors:** Chong Yang, Xuan Zhao, Zhi Luo, Lihu Wang, Mengjun Liu

**Affiliations:** 1 Hebei Agricultural University, College of Horticulture, Baoding, Hebei, China; 2 Hebei Agricultural University, Research Center of Chinese Jujube, Baoding, Hebei, China; 3 Hebei Agricultural University, National Engineering Research Center for Agriculture in Northern Mountaninous Areas, Baoding, Hebei, China; 4 Hebei University of Engineering, School of Landscape and Ecological Engineering, Handan, Hebei, China

**Keywords:** Chinses jujube, ZjSWEETs, Expression pattern, Abiotic stress

## Abstract

The novel sugar transporter known as SWEET (sugars will eventually be exported transporter) is involved in the transport and distribution of photosynthesis products in plants. The SWEET protein is also involved in pollen development, nectar secretion, stress responses, and other important physiological processes. Although SWEET genes have been characterized and identified in model plants, such as *Arabidopsis* and rice, little is known about them in jujube. In this study, the molecular characteristics of the SWEET gene family in the Chinese jujube (*Ziziphus jujuba* Mill.) and their expression patterns in different organs, at different fruit developmental stages, and under abiotic stress were analyzed. A total of 19 ZjSWEET genes were identified in jujube through a genome-wide study; these were classified into four sub-groups based on their phylogenic relationships. The gene structure analysis of ZjSWEET genes showed that all the members had introns. The expression patterns of different ZjSWEET genes varied significantly in different organs (root, shoot, leave, flower, fruit), which indicated that ZjSWEETs play different roles in multiple organs. According to the expression profiles by quantitative real-time PCR analysis during fruit development, the expression levels of the two genes (*ZjSWEET11*, *ZjSWEET18*) gradually increased with the development of the fruit and reached a high level at the full-red fruit stage. A prediction of the *cis*-acting regulatory elements indicated that the promoter sequences of ZjSWEETs contained nine types of phytohormone-responsive *cis*-regulatory elements and six environmental factors. In addition, the expression profiles by quantitative real-time PCR analysis showed that some of the ZjSWEETs responded to environmental changes; *ZjSWEET2* was highly induced in response to cold stress, and *ZjSWEET8* was significantly up-regulated in response to alkali and salt stresses. This study showed that the functions of the ZjSWEET family members of jujube are different, and some may play an important role in sugar accumulation and abiotic stress in jujube.

## Introduction

Carbohydrates are the basic energy-providing molecule in eukaryotes and are the main source of carbon (Redwan, Saidin & Kumar, 2016). They play an important role in the storage and transportation of plant nutrients, signal transduction, osmotic regulation, and stress resistance. They also directly or indirectly participate in many growth and development processes in plants, including the transport of various sugars, nitrogen intake, defense responses, and balancing various hormones (Lalonde, Wipf & Frommer, 2004). Leaves are typically the source of carbohydrates in plants. The transport of carbohydrates involves the movement of intercellular solution, which needs to pass through specific transporters. The accumulation of sugar in sink tissues like fruit mainly depends on the ability of source tissue to output photosynthetic products, the efficiency of phloem sugar transport, and the capability of the sugar transmembrane transport.

In the production process of plants, the balance between sink and source can be realized by adjusting the distribution and accumulation of sugars in source and sink, which promotes crop yield (Frank Baker, Leach & Braun, 2012). Sucrose is the main carbohydrate transported in higher plants. There are two forms of sucrose distribution: short-distance transportation and long-distance transportation (Sonnewald, 2011). As a relatively large polar solute, soluble sugars need corresponding transporters for transmembrane transport. Effective glucose transmembrane movement requires the operation of a variety of transporters (Buttner & Sauer, 2000), such as monosaccharide transporters (MST), sucrose transporters (SUT), and sugars will eventually be exported transporters (SWEET) (Sonnewald, 2011; Chen et al., 2012; Braun, 2012).

The SWEET gene family is a newly-identified sugar transporter gene family. It has the function of bidirectional sugar transport by promoting the diffusion of sugar across cell membranes or vacuole membranes along the concentration gradient (Frank Baker, Leach & Braun, 2012). The SWEET gene family is very conservative in evolution and exists widely in eukaryotes, animals, bacteria, fungi and archaea (Yuan & Wang, 2013). The membrane proteins encoded by SWEET genes have a certain number of conserved transmembrane domains, which are named MTN3/saliva (Frank Baker, Leach & Braun, 2012). These were first found in the nodulin in the root of *Medicago sativa* (Buttner & Sauer, 2000). SWEET gene family members have been identified in many plants recently, including 17 in *Arabidopsis*, 21 in rice, 29 in tomato, 52 in soybean, 18 in pear, 22 in apple, 27 in grape, 25 in banana, 68 in rape, 59 in wheat, 27 in sweet orange, and 25 in walnut. (Yuan & Wang, 2013; Chen et al., 2010; Chong et al., 2014; Patil et al., 2015; Feng et al., 2015; Li et al., 2017; Velasco et al., 2010; Miao et al., 2017; Miao et al., 2018; Gao et al., 2017; Gautam et al., 2019; Yao et al., 2021; Jiang et al., 2020).

The members of the SWEET gene family are involved in many physiological processes. *AtSWEET11* and *AtSWEET12* are sucrose transporters and are found in the phloem plasma membrane in *Arabidopsis*. Sucrose is the predominant form of carbon found in phloem tissue. *AtSWEET11* and *AtSWEET12* are localized to the *Arabidopsis* plasma membrane and are responsible for the efflux of intracellular sucrose into the cell wall space for loading into the phloem for long-distance transport of sucrose in plants (Chen et al., 2012). *MtSWEET11* is a nodule-specific sucrose transporter in *Medicago truncatula*, which is involved in the distribution of sucrose (Kryvoruchko et al., 2016). *AtSWEET9* is a nectary specific sugar transporter in dicotyledons with an important role in the production of nectar (Lin et al., 2014).

Plants are often subjected to various stresses that adversely affect their growth and development and may even cause death. These stresses include abiotic stresses (low temperature, alkali, and salt damage, *etc*.) and biotic stresses (pathogen infection, *etc*.). Under abiotic stress, plants have evolved a complex signaling system, and can alter many physiological, biochemical, and molecular processes through cellular and subcellular signaling pathways. Soluble sugars in plants play an important role under stress. They maintain the stability of the cellular osmotic pressure by regulating the distribution of sugars in various pathways in the body, allowing plants to grow in an orderly manner. According to relevant reports (Li et al., 2018; Zhou et al., 2018; Klemens et al., 2013; Qin et al., 2020), the SWEET gene families play different stress roles in different plants under abiotic stress. Under cold conditions, plants adapt by accumulating sugars in their vacuoles. For example, the *Atsweet11/12* double mutant in *Arabidopsis* exhibited greater cold tolerance than the wild type and two single mutants under cold stress (Hir et al., 2015). Zhao et al. (2018) found that *GhSWEET2aaat-Dt* and *GhSWEET3au2-DTt* were greatly induced under cold conditions in cotton and the expression of *GhSWEET2bu-DT* was significantly up-regulated. Salt stress caused by high concentration of Na^+^ and Cl^−^ also inhibits plant growth and development. Researchers found that *AtSWEET15* was significantly induced under high salinity conditions in *Arabidopsis*. Plants over-expressing *AtSWEET15* were highly sensitive to high salt stress, while mutant lines lacking *AtSWEET15* gene were less sensitive (Durand et al., 2016). Under high salt stress, both *BrSWEET11-LF* and *BrSWEET17-MF1* were significantly up-regulated in rapeseed. However, it is not known whether ZjSWEETs function in jujube trees under stress and this topic deserves further study. Chinese jujube (*Ziziphus jujuba* Mill.), a native fruit tree of China, is famous for its sugar-rich fruit and high resistance to abiotic stress, including drought, waterlogging, barrenness, salt, and alkali. The Chinese jujube is one of the oldest cultivated fruit trees in China and it has been introduced into at least 48 countries worldwide. The content of soluble sugar in the mature fruit of the Chinese jujube is higher than most other fruits (Liu, Liu & Liu, 2013). The stress resistance of fruit trees determines their ability to grow and develop properly and produce fruit. With the changes of environmental factors, the impacts of adversity stress on fruit trees are becoming more and more serious. The completion of jujube genome sequencing (Liu et al., 2014; Huang et al., 2016) made it possible to study the SWEET family at genome level. Recently, the analysis of sugar transport genes of the dried jujube cultivar ‘Junzao’ was reported, which provided insights into sugar transport genes related to differences in sugar accumulation between red and sour jujubes (Zhang et al., 2018). However, little is known about the SWEET gene family in the fresh jujube cultivar ‘Dongzao’. This study intends to identify the members of the SWEET gene family of the jujube genome to screen the key members related to sugar transport and to reveal the responses of the SWEET gene family members to abiotic stresses. This study will provide a theoretical basis for understanding the mechanism of sugar accumulation and molecular improvement of fruit quality in jujube.

## Materials & Methods

### Genome-wide identification of *SWEET* family genes in Chinese jujube

The protein sequences of *Arabidopsis* SWEETs (Chen et al., 2010) were used as the query sequences to identify the members of the SWEET gene family in Chinese jujube (*Ziziphus jujuba* Mill.). Based on the obtained *Arabidopsis* SWEET gene, BLASTP analysis was performed on the jujube genome database (https://www.ncbi.nlm.nih.gov/assembly/GCF_000826755.1) to determine candidate genes. The conservative domain analysis of the candidate members by the Conserved Domain Database (CDD) (Marchler-Bauer et al., 2009) and Pfam (http://pfam.xfam.org/) (Finn et al., 2006) confirmed the existence of MtN3/saliva domain in each member. The chromosomal positions, open reading frames (ORFs), and amino acid sequences were obtained from NCBI database. TMHMM Server v.2.0 (http://www.cbs.dtu.dk/services/TMHMM) and ProtParam in ExPASy were used to analyze the transmembrane region, isoelectric point (PI), and molecular weight of each member.

### Gene structure, multi-sequence alignment, and phylogenetic analyses

The exon/intron structure of the gene was analyzed using GSDS (http://gsds.cbi.pku.edu.cn/) (Guo et al., 2007). The conserved motifs of SWEETs protein were analyzed using online MEME tools (https://meme-suite.org/meme/tools/meme). The MEME parameter settings were as follows: the number of motifs was 10, and the length of motifs was 5 to 50.

**Figure 1:**
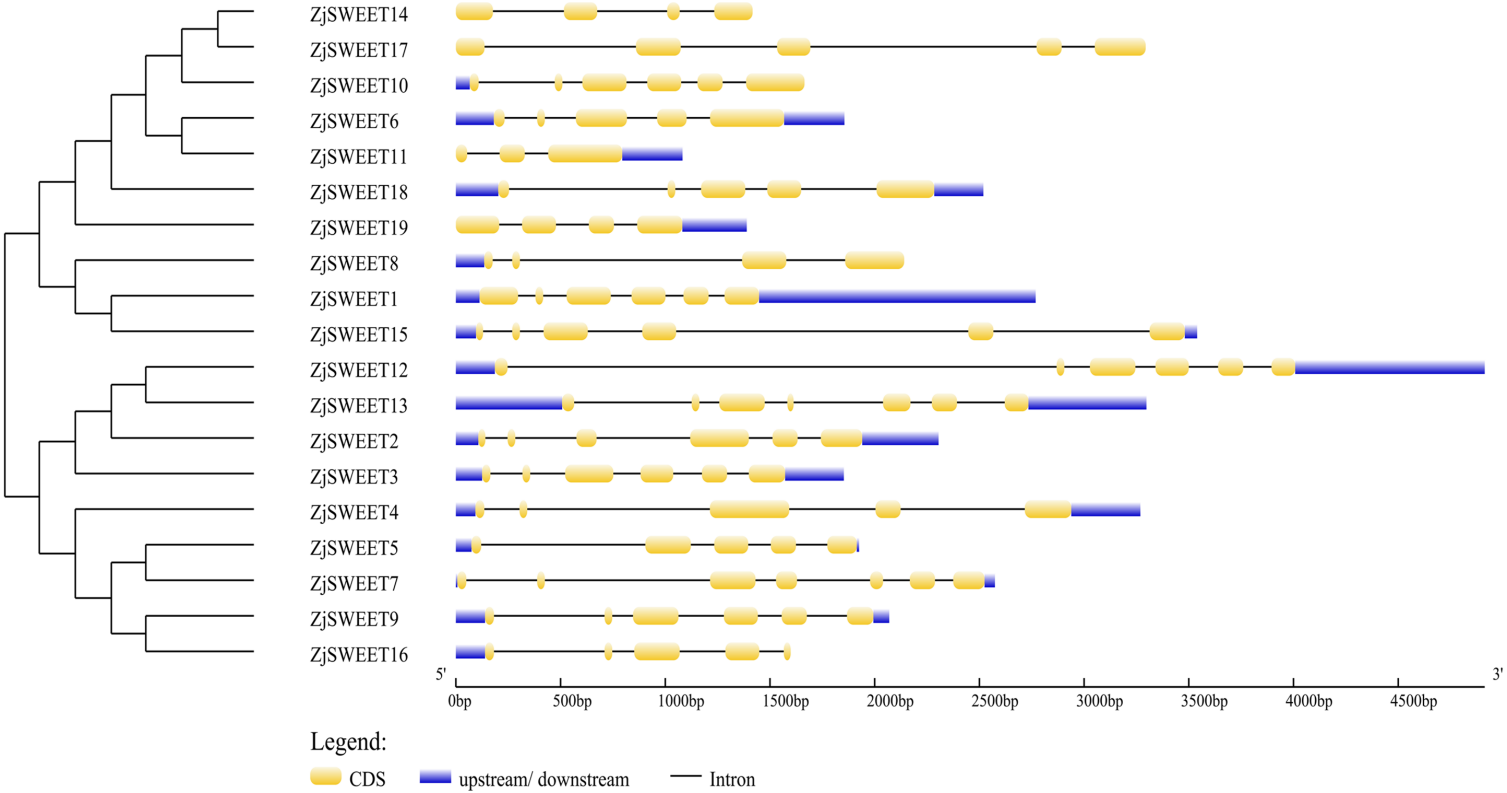
Schematic diagrams of the gene structures of ZjSWEETs. The yellow, blue boxes and the black lines indicated the exons, UTRs and introns, respectively.

**Figure 2:**
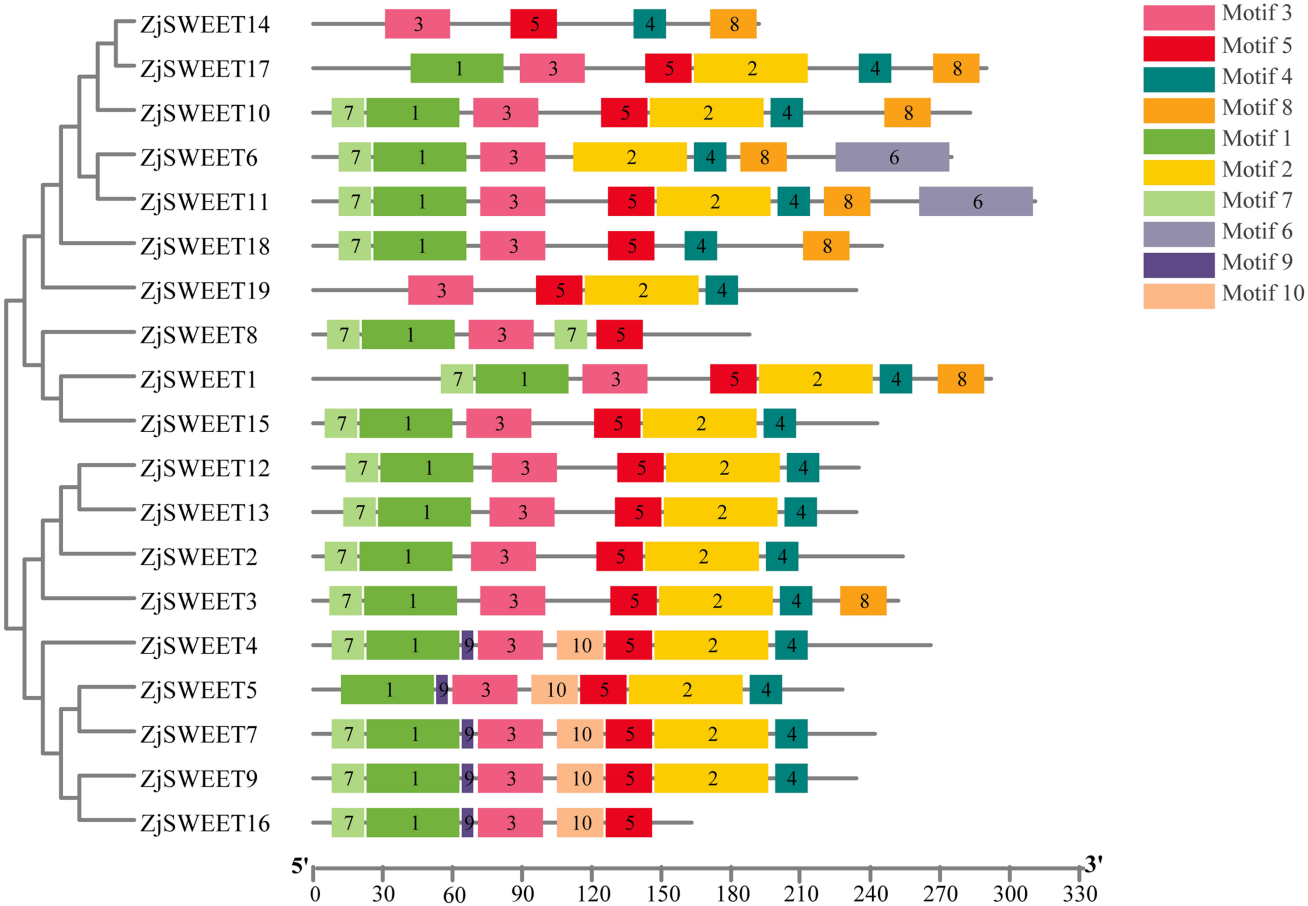
The motif compositions of ZjSWEETs in corresponding to the phylogenetic tree. The phylogenetic tree was constructed using maximum-likelihood based algorithms. Conserved motifs of ZjSWEET proteins were detected using the online MEME program and mapped using TBtools software. Motifs 1–10 are displayed in differently colored boxes. Sequence information for each motif is provided in File S3. The length of the protein can be estimated using the scale at the bottom.

The amino acid sequences of 17 *Arabidopsis thaliana* SWEET genes, 20 *Malus domestica* SWEET genes (Velasco et al., 2010; Wei et al., 2014) and 19 jujube SWEET genes were used to construct an unrooted phylogenetic tree. SWEET proteins from *Arabidopsis thaliana* and *Malus domestica* were downloaded from the NCBI (https://www.ncbi.nlm.nih.gov/) (File S4). The amino acid sequences of the members of SWEET gene family were analyzed by ClustalW of MEGA5.2. The parameters of alignment were as follows: gap opening penalty, 10.00; gap extension penalty, 0.20 (both in pairwise alignment and in multiple alignments); protein weight matrix, gonnet; residue-specific penalties, on; hydrophilic penalties, on; gap separation distance, 4; end-gap separation, on; use negative matrix, off; and delay divergent cutoff (%), 30. Phylogenetic trees were constructed by the maximum-likelihood based algorithms of MEGA5.2. The parameters chosen for the constructed trees were as follows: statistical method, maximum-likelihood; test of phylogeny, bootstrap method; number of bootstrap replications, 1,000; substitution types, amino acid; model/method, Jones-Taylor-Thornton (JTT) model; rates among sites, gamma distributed; ML heuristic method, nearest-neighbor-interchange; initial tree for ML, make initial tree automatically (Tamura et al., 2011). The analysis of 19 ZjSWEET genes in Chinese jujube and the construction of the evolutionary tree are the same as above (Figs. 1 and 2).

### *Cis* -element analysis of putative promoters

The 2 Kb base sequences were taken from the NCBI database at the upstream position of the transcription start sites to form the promoter sequences of each ZjSWEET gene. The promoter sequences were input into the PlantCare (https://bioinformatics.psb.ugent.be/webtools/plantcare/html/) online tool to analyze their *cis*-acting elements (Lescot et al., 2002).

### Plant materials and treatments

The tissue materials for specific gene expression analysis were obtained from the root, stem, leaf, flower, and fruit of the plants. All samples were collected from three different sampling trees and were repeated three times.

The fruits used to analyze gene expression in different developmental stages were taken from the young fruit stage (Y), early white mature fruit stage (EWM), white mature stage (WM), half-red fruit stage (HR), and full-red fruit stage (FR) of *Ziziphus jujuba* Mill. ‘Linyilizao’ and ‘Beijingjidanzao’, which were planted in the national jujube germplasm repository in the Shanxi province. Among the different varieties of jujube, ‘Beijingjidanzao’ has a higher sugar content, and ‘Linyilizao’ has a lower sugar content. These trees were planted in the same garden under the same cultivation conditions.

The material used to investigate the response of ZjSWEETs to the abiotic and biotic stresses was the callus of *Ziziphus jujuba* Mill. Guanyangchangzao. The calluses were placed at 4 °C for cold treatment and at 25 °C for the control. The callus tissues were subjected to 150 mM NaCl and NaHCO_3_-NaOH solution (pH 9.5) for salinity and alkaline treatments. All controls were kept in an aqueous solution (Guo et al., 2017; Wang et al., 2020). The treated and control materials were collected at 0 h, 1 h, 6 h, and 24 h after treatment, respectively, and all of the collected samples were immediately stored in liquid nitrogen for next use.

### RNA isolation and qRT- PCR analysis

The above materials include jujube fruits at different development stages, callus tissues treated with low temperature, alkali, salt, and their corresponding controls were used for RNA extraction. The total RNA was extracted using the RNA prep Pure Plant Kit (Tiangen Biotech, Beijing, China) according to the manufacturer’s protocol, then the DNA in total RNA was removed with DNase I (Tiangen Biotech, Beijing, China). The concentration and purity were checked using the NanoDrop2000 spectrophotometer. First-strand cDNA synthesis with reverse transcriptase (Tiangen Biotech, Beijing, China) according to the manufacturer’s protocol using 1 µg of RNA template.

The expression analysis was carried out by quantitative reverse-transcription PCR (qPCR) on the Bio-Rad iQ™5 using TransStart Top Green qPCR SuperMix AQ131 (TransGen Biotech, Beijing, China). The entire reaction system measured 20 µL and was comprised of 10 µL of 2 ×SYBR Premix ExTaq™, 0.4 µL each of 10µM primers, 1 µL diluted cDNA, and 8.2 µL ddH_2_O. The conditions were: 3 min at 95 °C, followed by 40 cycles of 5 s at 95 °C, 15 s at 55–62 °C and 15 s at 72 °C. Three biological replicates were performed for analysis. Relative expression levels of ZjSWEETs were calculated according to the 2^−ΔΔCt^ method (Livak & Schmittgen, 2003). The *ZjACT* was used as a reference gene (Bu, Zhao & Liu, 2016). The primers were listed in File S6 designed by using Primer 5.

### Gel semi-quantification

Gel bands were quantified by using ImageJ software. The ACT from each tissue (root, stem, leaf, flower, fruit) was used as a control to calculate the bands of each gene.

### Heatmap construction

The expression profiles of all ZjSWEETs in different developmental stages were taken from the young fruit stage (Y), early white mature fruit stage (EWM), white mature stage (WM), half-red fruit stage (HR), and full-red fruit stage (FR) of *Ziziphus jujuba* Mill. ‘Linyilizao’ and ‘Beijingjidanzao’ are illustrated by a colour gradient heatmap. The heatmap was constructed by TBtools (Chen et al., 2020), and cluster rows were selected.

The expression profiles of all ZjSWEETs in response to low temperature, alkaline, and salt stresses are illustrated by a colour gradient heatmap. The heatmap was constructed by TBtools. The values shown in the heat map were: experimental group/ 0 h calluses.

## Results

### Genome-wide identification of ZjSWEETs in Chinese jujube

ZjSWEET genes were excavated based on the sequenced genomes of ‘Dongzao’. The homology search and conservative domain analysis were combined to identify SWEET genes in the jujube genome. A total of 19 SWEET genes were found. The members of the jujube SWEET gene family were named *ZjSWEET1*-*19* referring to *Arabidopsis thaliana*. Their amino acid length, protein molecular weight, isoelectric point, and chromosome localization were forecasted and analyzed (Table 1). The amino acid length (aa) encoded by the members of the SWEET gene family in jujube ranged from 163 to 292, of which 15 genes encode more than 200 amino acids (Table 1). The molecular weight of SWEET protein varied from 18.2 to 33 kDa, and the range of protein isoelectric point varied from 4.88 to 9.84. The chromosome locations of the SWEET genes in jujube were analyzed and were found to be distributed on chromosomes 1, 2, 3, 4, 5, 7, 11 and 12 of the jujube genome, while 6 ZjSWEETs (*2*, *9*, *10*, *11*, *16*, *17*) were not localized in the assembled regions.

**Table 1:** The information of ZjSWEET gene family in Chinese jujube.

Gene name	NCBI Reference sequence	Introduction	Chromosomes	Position	ORF (bp)	Size (aa)	MW (KD)	TMs	PI
*ZjSWEET1*	XM_016019564.1	bidirectional sugar transporter SWEET17-like (LOC107411894)	Chr2	21108341- 21110073+	878	292	33.0	6	8.47
*ZjSWEET2*	XM_016047612.1	bidirectional sugar transporter SWEET1 (LOC107435979)	Unplaced Scaffold	80450- 82795+	764	254	28.3	7	9.62
*ZjSWEET3*	XM_016022717.1	PREDICTED: bidirectional sugar transporter SWEET3 (LOC107414580)	Chr3	23901230- 23903077-	758	252	28.7	7	8.86
*ZjSWEET4*	XM_016040575.1	bidirectional sugar transporter SWEET4 (LOC107429824)	Chr11	601832- 605393+	800	266	29.3	7	9.66
*ZjSWEET5*	XM_025076021.1	bidirectional sugar transporter SWEET5-like (LOC107423593)	Chr7	27569657- 27571791+	686	228	26.0	7	9.15
*ZjSWEET6*	XM_016026756.1	bidirectional sugar transporter SWEET12-like (LOC107418084)	Chr5	7307509- 7309366-	827	275	31.0	6	7.62
*ZjSWEET7*	XM_016023740.1	bidirectional sugar transporter SWEET6b (LOC107415419)	Chr4	3126613- 3129448+	728	242	27.0	7	9.44
*ZjSWEET8*	XM_016019510.1	bidirectional sugar transporter SWEET16-like (LOC107411840)	Chr4	21027146- 21029285+	566	188	20.8	5	9.84
*ZjSWEET9*	XM_016012790.1	bidirectional sugar transporter SWEET5-like (LOC107405701)	Unplaced Scaffold	28790- 31346+	704	234	26.3	6	8.46
*ZjSWEET10*	XM_016011459.1	PREDICTED: bidirectional sugar transporter SWEET10-like (LOC107404506)	Unplaced Scaffold	84150- 86090+	851	283	32.3	7	8.53
*ZjSWEET11*	XM_016011458.1	PREDICTED: bidirectional sugar transporter SWEET15-like (LOC107404505)	Unplaced Scaffold	69796- 70875+	527	175	19.6	7	6.22
*ZjSWEET12*	XM_016043781.1	PREDICTED: bidirectional sugar transporter SWEET2-like (LOC107432598)	Chr12	8987461- 8992375+	707	235	26.3	7	9.03
*ZjSWEET13*	XM_016020790.1	PREDICTED: bidirectional sugar transporter SWEET2a-like (LOC107412938)	Chr1	5902810- 5905851-	704	234	26.3	7	4.88
*ZjSWEET14*	XM_016026320.1	PREDICTED: bidirectional sugar transporter SWEET14-like (LOC107417685)	Chr5	3340092- 3341508-	578	192	21.5	4	7.63
*ZjSWEET15*	XM_016044393.1	bidirectional sugar transporter SWEET17 (LOC107433140)	Chr12	17546397- 17549966-	731	243	26.6	7	6.09
*ZjSWEET16*	XM_016012791.1	PREDICTED: bidirectional sugar transporter SWEET5-like (LOC107405702)	Unplaced Scaffold	26686-28290+	491	163	18.2	4	9.37
*ZjSWEET17*	XM_016011343.1	PREDICTED: bidirectional sugar transporter SWEET14-like (LOC107404397)	Unplaced Scaffold	71405- 74889+	872	290	33.1	6	9.33
*ZjSWEET18*	XM_025073303.1	PREDICTED: Ziziphus jujuba bidirectional sugar transporter N3-like (LOC107417626)	Chr5	3065280- 3067798-	737	245	27.8	5	9.55
*ZjSWEET19*	XM_025067594.1	PREDICTED: Ziziphus jujuba bidirectional sugar transporter NEC1-like (LOC107434690)	Unplaced Scaffold	49580- 50968+	704	234	26.6	6	6.43

### Gene structure and motif analysis

In order to further understand the biological function of the members of the SWEET gene family, the number and location of introns and exons of the 19 genes were analyzed using the online GSDS (Fig. 1). The comparative analysis of the coding sequence and genome sequence of jujube SWEET family members showed that all the gene sequences of the members contained introns. Most ZjSWEETs contained four to five introns; *ZjSWEET4*, *5*, *6*, *16*, *17*, and *18* contained four introns; *ZjSWEET1*, *9*, *10*, *12*, *15* contained five introns; *ZjSWEET3*, *8*, *14* contained three introns; *ZjSWEET7*, *13* contained six introns; and *ZjSWEET11* contained only two introns.

In eukaryotes, SWEET proteins have seven transmembrane *α*-helical domains, 7-TMs, which consist of tandem repeats of two 3-TMs units separated by a TM unit, containing a functional transporter consisting of at least four TMs (Chen et al., 2010; Livak & Schmittgen, 2003). To confirm the presence of the transmembrane domain, the protein sequences of ZjSWEETs were submitted to the HMMER online website, as shown in Table 1. The results showed that 10 ZjSWEETs contained seven TMs, five ZjSWEETs contained six TMs, while the rest had 5 TMs (*ZjSWEET8*, *18*), or four TMs (*ZjSWEET14*, *16*) (Table 1). All the ZjSWEETs had two MtN3/saliva domains except *ZjSWEET8* and *18* which had only one (Table 1). Furthermore, 10 motifs in ZjSWEETs were predicted using the MEME database (Fig. 2). Motif analysis found that motif 3(LVITINSIGCVIETIYIAJFLIYAPKKKR) was present in all jujube SWEET proteins, motif 1 was absent only in ZjSWEET14 protein, and motif 5 was absent only in ZjSWEET6 protein. Motif 4 was present in other proteins except the ZjSWEET8 protein and ZjSWEET16 protein, and motif 6 was only present in the ZjSWEET6 and ZjSWEET11 proteins.

### Sequence alignments and phylogenetic analysis

**Figure 3:**
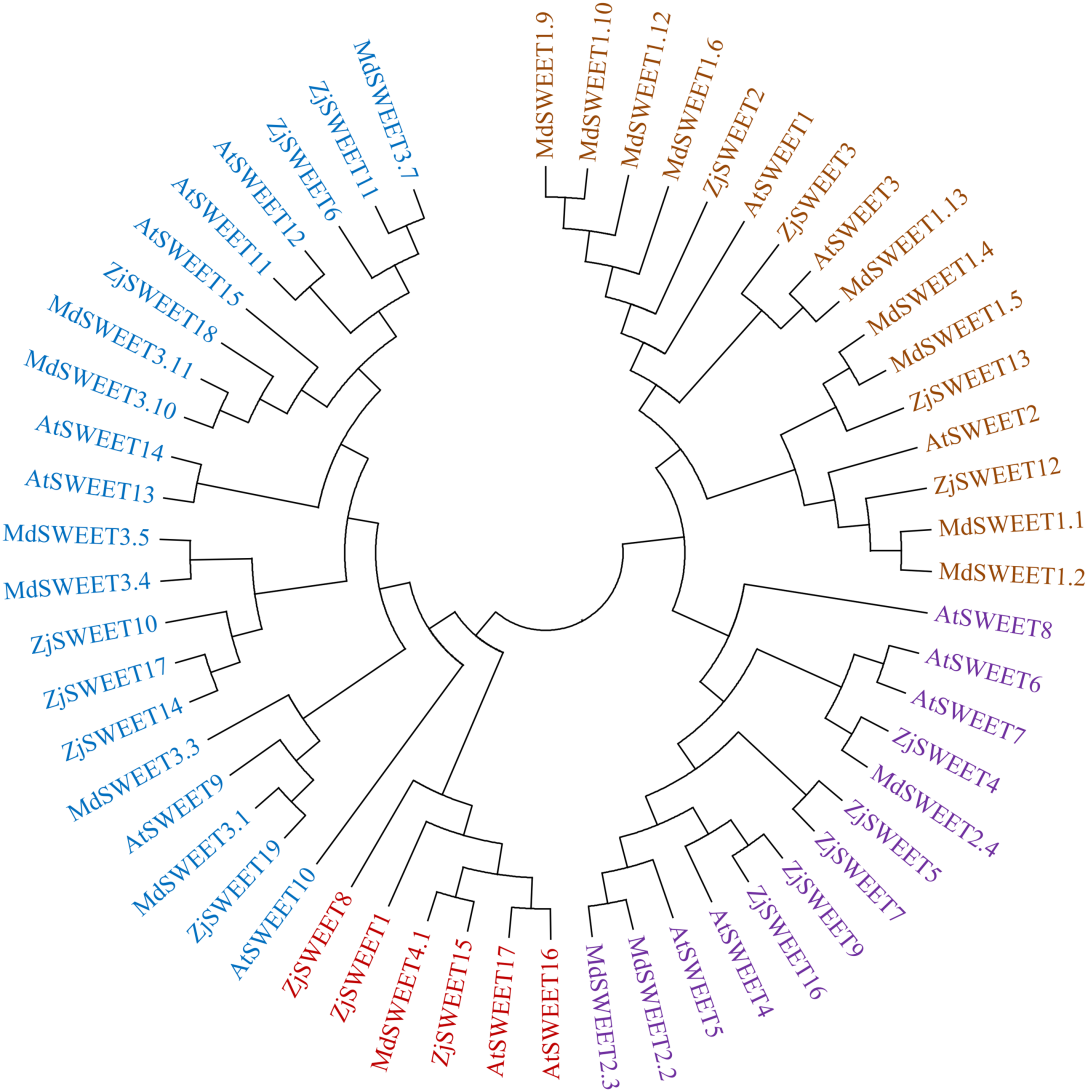
The phylogenetic analysis of ZjSWEETs, MdSWEETs and AtSWEETs protein sequences. MEGA 5.2 was used to construct the phylogenetic tree with the maximum-likelihood method.

To better understand the evolutionary origin and function of jujube SWEET gene, a phylogenetic tree was computed by using the full-length sequence of jujube SWEET protein with *Arabidopsis thaliana* and *Malus domestica* to comprehend their phylogenetic relationship. The results showed that the 19 jujube SWEET genes could be divided into four groups according to previously reported classes for *Arabidopsis thaliana*. In detail, the four subfamilies were named class I-IV, which respectively contained four, five, seven and three SWEET genes (Fig. 3). Four ZjSWEETs (*ZjSWEET2*, *3*, *12*, *13*), 3 AtSWEETs (*AtSWEET1*-*3*) and 9 MdSWEETs (*MdSWEET1.1*, *1.2*, *1.4*, *1.5*, *1.6*, *1.9*, *1.10*, *1.12*, *1.13*) were clustered in class I, while 5 ZjSWEETs (*ZjSWEET4*, *5*, *7*, *9*, *16*), 5 AtSWEETs (*AtSWEET4*-*8*), and 3 MdSWEETs (*MdSWEET2.2*, *2.3*, *2.4*) belonged to class II. Class III contained 7 ZjSWEETs (*ZjSWEET6*, *10*, *11*, *14*, *17*, *18*, *19*), seven AtSWEETs (9–15), six MdSWEETs (*MdSWEET3.1*, *3.3*, *3.4*, *3.5*, *3.10*, *3.11*). A total of three ZjSWEETs (*ZjSWEET1*, *8*, *15*), two AtSWEETs (*AtSWEET16*, *17*), and *MdSWEET4.1* were included in class IV.

### Prediction of *cis*-acting regulatory elements

It has been reported that the SWEET gene family plays important roles in the biotic and abiotic stress responses of plants (Seo et al., 2011; Li et al., 2018; Zhou et al., 2018; Klemens et al., 2013; Qin et al., 2020). We analyzed the *cis*-regulatory elements of the upstream sequence located 2 Kb from the ATG codon to reveal the function of ZjSWEETs. Promoter sequence analysis by PlantCARE revealed different *cis*-elements (Table 2). The results showed that the promoter sequences of ZjSWEETs contained 9 types of phytohormone-responsive *cis*-regulatory elements and 6 environmental factors, including ABRE, the CGTCA and TGACG motifs, ERE, the GARE motif, P-box, TATC-box, TCA element, TGA element, ARE, LTR, MBS, TC-rich repeats, WUN-motif, and Circadian-motif, suggesting that ZjSWEET genes may be involved in diverse stress responses. Among them, the promoter region of *ZjSWEET8* contained 10 types of phytohormone responsive *cis*-elements, while *ZjSWEET3* and *ZjSWEET17* contained the least types. The promoters of the 19 ZjSWEETs contained at least five phytohormone-responsive *cis*-elements (*ZjSWEET17*), while the promoter of *ZjSWEET8* contained the most (24 *cis*-elements). The aerobic-responsive *cis*-element (ARE) was present in all the ZjSWEET genes, suggesting important roles for these genes in anaerobic stress responses. The ABRE motif is involved in the ABA response and was included in all the promoters of the 19 ZjSWEETs except *ZjSWEET4* and *ZjSWEET5.* These data suggest that ZjSWEETs may be involved in the response to environmental stress through a complex mechanism, and that each ZjSWEET gene can be induced by different environmental stresses.

**Table 2:** Predicted *cis*-acting element in 2kb upstream regions of ZjSWEETs.

	ABA	MeJA		Eth- ylene	Gibberellin			Salicylic acid	Auxin	Ana- erobic	Low- Tem- perature	Dro- ught	Defense and stress	Wound	Circadian control	Total
	ABRE	CGTCA -morif	TGACG -motif	ERE	GARE- motif	P- box	TATC- box	TCA- element	TGA- element	ARE	LTR	MBS	TC-rich repeats	WUN- motif	Circadian	
ZjSWEET1	2			3	1		1	1		1	1				1	11
ZjSWEET2	3	1	1	1					1	3			1	1		12
ZjSWEET3	3								1	3				1		8
ZjSWEET4				3		2		3		3		1				12
ZjSWEET5				4		1			1	2		1		1		10
ZjSWEET6	5	1	1							3	1		1	1	1	14
ZjSWEET7	6	1	1		1	1		4		4			2		1	21
ZjSWEET8	2	3	3	2		1		2	1	4	1			4	1	24
ZjSWEET9	4	2	2					2		2	1	2		1		16
ZjSWEET10	2	1	1	2						6	1		1	2	1	17
ZjSWEET11	5	1	1					1		3	1		1	1		14
ZjSWEET12	2			1	1	1	1		1	3	2	2		1	2	17
ZjSWEET13	5							2		2	1		1			11
ZjSWEET14	4			6				2		2	1					15
ZjSWEET15	2			2		1		1		4				1		11
ZjSWEET16	5	3	3	1				1		2	1	1	1	1		19
ZjSWEET17	2							1		1				1		5
ZjSWEET18	5	1	1	3				2		2						14
ZjSWEET19	2	2	2	1		1			1	4	1	1		1		16

### Expression profiles of ZjSWEETs in various organs and different developmental stages of fruit

**Figure 4:**
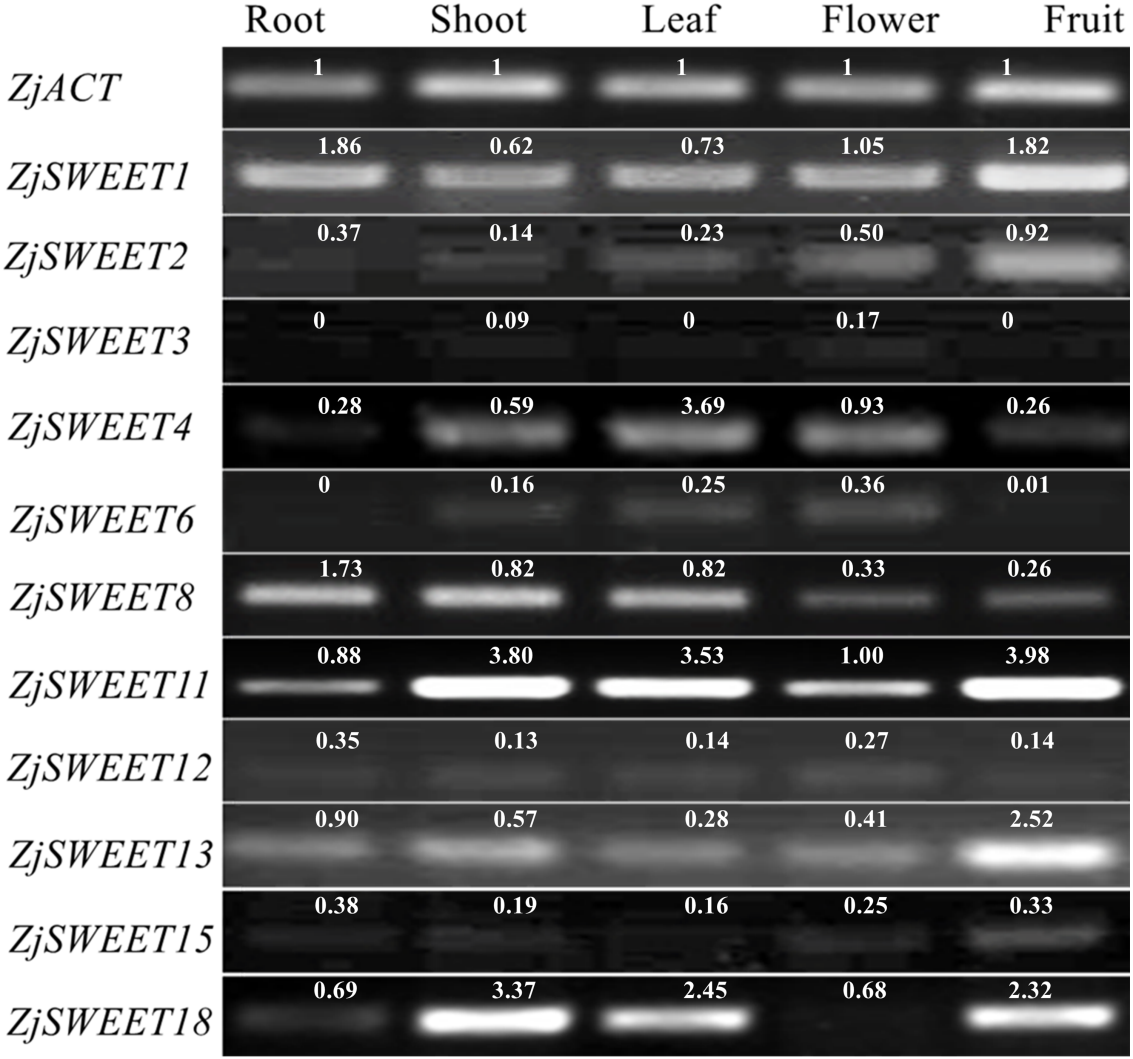
Expression patterns of ZjSWEETs in five tissues of jujube by RT-PCR. *ZjACT* was used as an internal control. The gel bands with software ImageJ. The ACT from each tissue (root, stem, leaf, flower, fruit) was used as a control to calculate the bands of each gene.

qPCR testing was used to determine their expression patterns in five plant organs (root, shoot, leave, flower, fruit) of ‘Dongzao’ to investigate the expression profiles of the jujube SWEET genes. The qPCR signal for *ZjSWEET5*, *7*, *9*, *10*, *14*, *16*, *17*, and *19* was not detected in the five tissues of jujube suggesting that they may not be expressed, or that the expression level is low. Other genes were expressed multiple tissues, but their expression levels varied considerably (Fig. 4). For example, *ZjSWEET1* and *ZjSWEET11* were highly expressed in all organs, while *ZjSWEET3*, *ZjSWEET6* and *ZjSWEET12* were hardly expressed in fruit. *ZjSWEET18* had very low expression in flowers, but high in stems, leaves, and fruit tissues. These results showed that different ZjSWEET genes had different tissue-specific expression patterns, which indicated that ZjSWEETs play different roles in multiple organs.

**Figure 5:**
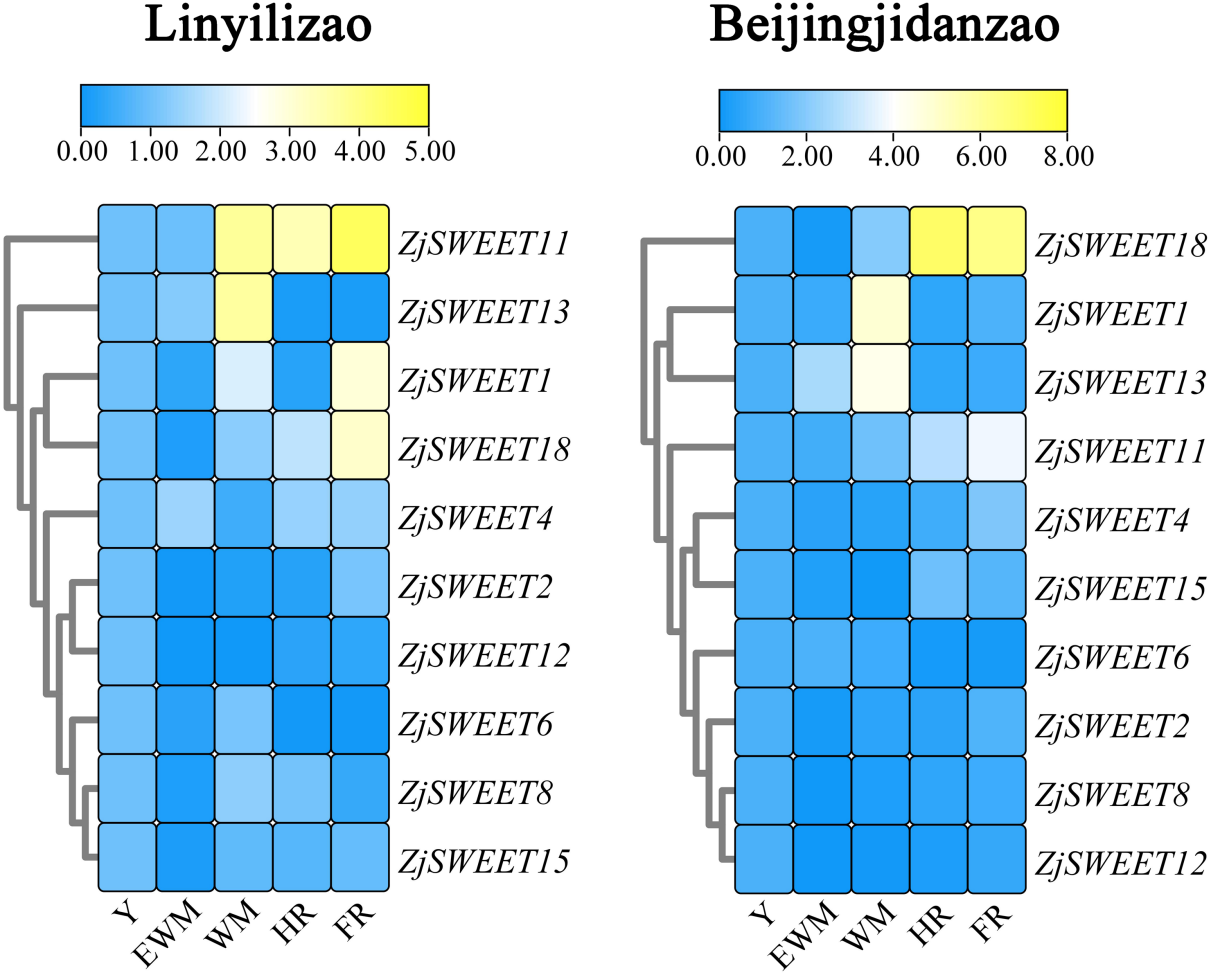
Heat maps of the relative expression of ZjSWEETs during fruit ripening in ‘Linyilizao’ and ‘Beijingjidanzao’. Y, young fruit; EWM, early white mature fruit; WM, white mature fruit; HR, half-red fruit; FR, full red fruit. Scaled log2 expression values based on qRT-PCR data are shown from blue to yellow, indicating low to high expression.

Existing studies have shown that *ZjSWEET5*, *7*, *9*, *14*, *16*, *17*, and *19* had little expression in jujube fruits (Liu et al., 2014). In order to understand the expression of sugar transporter genes in jujube, the famous high sugar variety ‘Beijingjidanzao’ and low sugar variety ‘Linyilizao’ were used. As shown in Fig. 5, the expression patterns of some ZjSWEETs during fruit development were consistent in ‘Linyilizao’ and ‘Beijingjidanzao’, in which the expression levels of *ZjSWEET11* and *ZjSWEET18* increased with fruit ripening and reached higher levels in the half-red fruit stage (HR) and full-red fruit stage (FR). The expression level of *ZjSWEET13* gradually reached its highest level in the white mature stage (WM), and decreased with the fruit ripening. However, the expression levels of the remaining few genes were different between the two jujube varieties. The expression level of *ZjSWEET1* in ‘Linyilizao’ was the highest at the ripening stage of fruit, but it was the highest in white mature stage (WM) in ‘Beijingjidanzao’. The expression of *ZjSWEET4*, *11*, *18* increased along with the ripening of the fruit; the expression of *ZjSWEET11* and *ZjSWEET18* increased to a very high level at the whole red stage. However, the expression of some genes (*ZjSWEET8*, *12*, *13*, *15*) decreased with the development of fruit. The expression of *ZjSWEET2* was higher at the young fruit stage and full red fruit stage than that in the white mature stage.

### Expression profiles of ZjSWEETs under abiotic stresses

**Figure 6:**
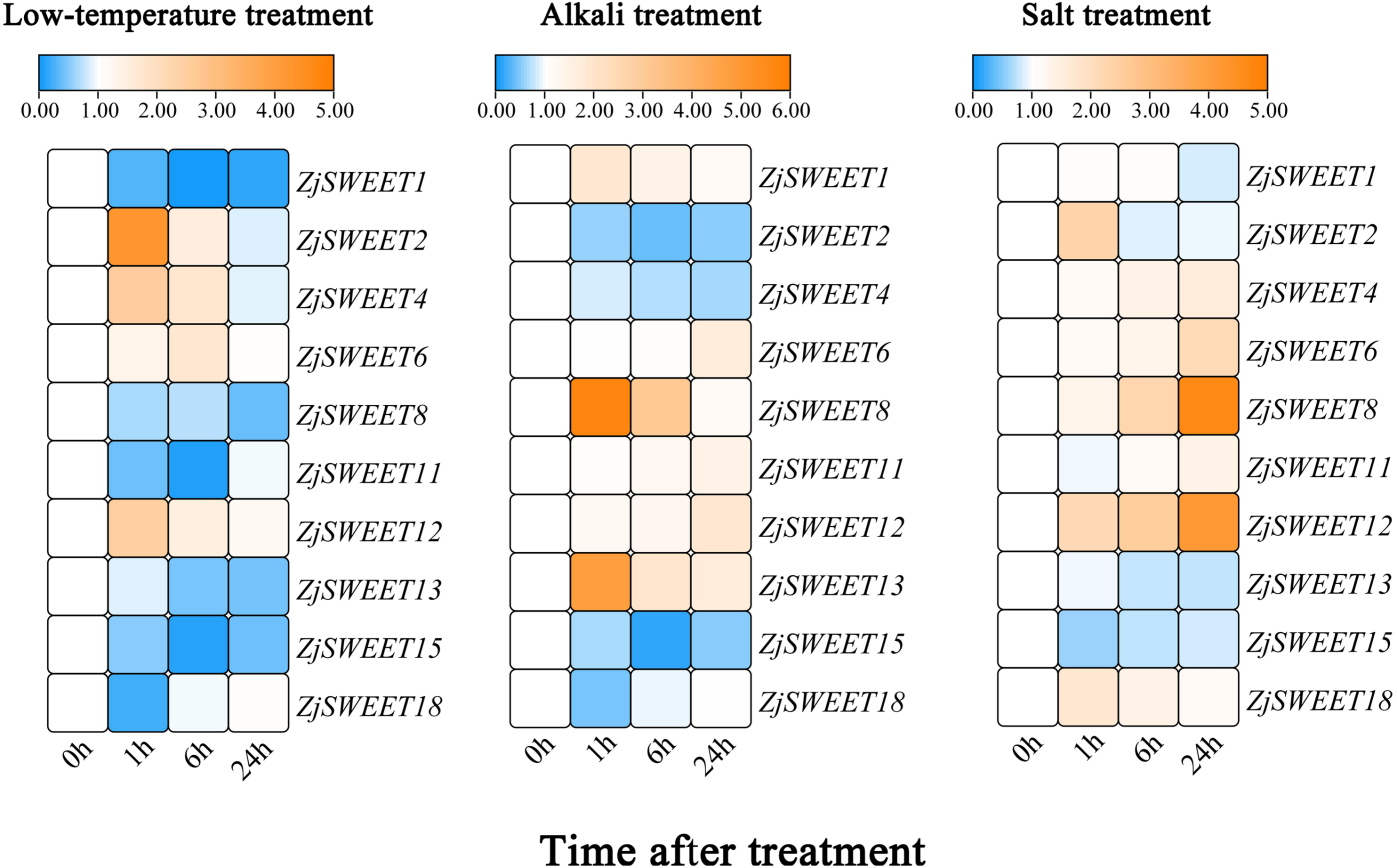
Relative expression profiles of *ZjSWEETs* in response to abiotic stresses. Heatmap analysis of the relative expression profiles of ZjSWEETs in response to abiotic stresses. Different colours indicate that the corresponding gene was significantly up- or downregulated at different time points. From left to right: low-temperature treatment, alkaline treatment and salt treatment. The material was the callus of *Ziziphus jujuba* Mill*.* ‘Guanyangchangzao’.

In order to verify the sugar transport gene under stress, the callus of high sugar variety ‘Guanyangchangzao’ was selected for verification. We detected the expressions of ZjSWEETs in low temperature conditions to determine its role under abiotic stress. The results showed that all of the ZjSWEET genes were downregulated except *ZjSWEET2*, *4, 6*, and *12* (Fig. 6). Compared with the control, the expression level of *ZjSWEET18* decreased and reached the lowest value at 1 h (about 0.2 times), while the expression levels of *ZjSWEET1*, *11*, and *15* reached the lowest value at 6 h. The expression of *ZjSWEET2*, *12*, and *4* increased at 1 h, but *ZjSWEET6* was induced at 6 h. Therefore, there may be different mechanisms for these ZjSWEETs involved in the response of jujube to low temperature stress.

In addition, these genes all responded to alkaline stress (Fig. 6). Most of the ZjSWEET genes were up-regulated except *ZjSWEET2*, *4*, *15* and *18* after alkaline treatment. For example, *ZjSWEET1, 8*, and *13* were highly increased at 1 h, while *ZjSWEET6* and *12* were enhanced at 24 h. This indicates that ZjSWEETs have a different mechanism in the tolerance of jujube alkali.

We also found that there were differences in the expression of different ZjSWEETs under salt stress, among which the expression of *ZjSWEET1*, *13*, and *15* were down-regulated, while *ZjSWEET8* and *12* were highly induced (Fig. 6). We also made a significant analysis of the data, as shown in File S8.

Under low temperature, alkali, and salt stress, the expressions of *ZjSWEET12* all increased, indicating that *ZjSWEET12* may play an important role in resistance to various stresses. In summary, ZjSWEET genes were induced or repressed by the above treatments indicating their indispensable regulation role in adapting abiotic stresses of jujube.

## Discussion

The SWEET transporter contains 2 MtN3/saliva trans-membrane domains and seven trans-membrane *α*-helices in eukaryotes (Chen et al., 2010). In this study, a bioinformatic approach identified 19 SWEET genes in the ‘Dongzao’ jujube genome. Relative to the 21 SWEET genes in the ‘Junzao’ genome (Zhang et al., 2018), we analyzed the corresponding genes of these two extra genes in the ‘Dongzao’ and found that they did not have typical institutional characteristics, even a TM. Therefore, they were not listed as candidate genes. These results are consistent with previously published research reporting 17 SWEET genes in *Arabidopsis* (Chen et al., 2010) which all share a conserved domain, indicating that the SWEET family remains conservative in evolution. ZjSWEET proteins contain the MtN3_saliva domain, all of them have at least one *α*-helix transmembrane domain and most contain seven *α*-helix transmembrane domains. According to previous findings in *Arabidopsis*, phylogenetic analysis discriminated four branches in the evolution of ZjSWEETs and the number of genes in each branch is coherent with that in *Arabidopsis*. The results showed that there was a great relationship between the relative selection of monosaccharides and disaccharides in different branches of the SWEET protein (Eom et al., 2015). The SWEET proteins of branches I and II were mainly responsible for the transport of hexose, the SWEET proteins of branch III were mainly responsible for the transport of sucrose, and the SWEET proteins of branch IV were expressed in the vacuole of *Arabidopsis* and were mainly responsible for the transport of fructose.

The content of soluble sugar (mainly sucrose, glucose, and fructose) is an important index to determine the quality of fruit. The SWEET gene family may play a key role in fruit development. It is reported that the SWEET family is involved in the fruit development and ripening process of grapes, apples, and other plants (Chong et al., 2014; Jian et al., 2016; Wei et al., 2014), but the specific molecular mechanism of how the SWEET family is involved in fruit development and ripening is not clear. Seven SWEET genes in the *Citrus sinensis* genome were highly expressed in the fruit (Zheng et al., 2014), and the expression of 6 SWEET genes was enhanced with the development of the berry in grape (Chong et al., 2014). The expression of *MdSWEET1.1*/*2*, *MdSWEET2.4* and *MdSWEET3.5* in apple was higher in young fruit, but *MdSWEET3.6*/*7* was more abundant in mature fruit (Wei et al., 2014). Some studies of tomato have also found that the expression of SWEET changed greatly during fruit development and ripening (Feng et al., 2015). The content of soluble sugar in the mature fruit of jujube is higher than most fruits; the soluble sugar content of fresh jujube is 25%–30% (Yang, Wang & Pan, 2009; Liu & Wang, 2009). Soluble sugar is one of the dominant nutrients of jujube, and it is also the main nutrient and flavor substance of most fruits. Sucrose, glucose, and fructose are the main sugars of the jujube fruit. The sugars accumulate with the development of the jujube fruit. As sugar transporters, SWEET proteins may play vital roles in sugar distribution and accumulation during fruit development. In this study, 10 ZjSWEET genes were found to express in jujube fruit, among them, the expression of *ZjSWEET2*, *11*, and *18* are the highest during the ripening stage of the fruit. The expression of *ZjSWEET11* and *18* increased several times with the ripening of fruit, which suggested that *ZjSWEET11* and *18* may be involved in the transportation and distribution of soluble sugar in jujube. Therefore, further studies on sugar distribution and accumulation may provide useful information for improved fruit quality and yield in the jujube.

Plants maintain the balance of cell osmotic potential by regulating the redistribution of soluble sugar *in vivo* under abiotic stress to keep sustainable growth (Yamada et al., 2010). Sugar transporters are key to regulating the redistribution of soluble sugar. These transporters can respond to a variety of stresses and are closely related to the adaption of plants to stress. During the cold acclimation of *Camellia sinensis*, the expression of *CsSWEET2*, *3*, and *16* was significantly reduced, while the expression of *CsSWEET1* and *CsSWEET17* increased dramatically (Yue et al., 2015). In conditions of high sugar, high salt, and high and low temperatures, the expression of several SWEET genes in tomato leaves, roots, green fruits, and red fruits changed significantly (Feng et al., 2015). The current research on the response mechanism of the AtSWEET gene to abiotic stress in *Arabidopsis* is significant. *AtSWEET17* was responsible for the two-way transportation of fructose to maintain the balance of fructose in the cytoplasm of *Arabidopsis* leaves and roots. It was also involved in an adaption response to abiotic stress such as low nitrogen and cold stress (Guo et al., 2014; Chardon et al., 2013). The transcript level of *AtSWEET16* decreased under low temperature, drought and low nitrogen stress. The over-expression of *AtSWEET16* in *Arabidopsis thaliana* significantly improved the seed germination rate, and frost resistance, indicating that SWEET family members are involved in the stress response process (Li et al., 2018). In this study, we found that most ZjSWEETs were expressed in response to abiotic stress, and the expression patterns of the same gene were different in different treatments. The members of ZjSWEET gene family may act as sugar transport carriers to participate in sugar transport as sugar transport carriers and may change the osmotic pressure of the plant and improve its adaptability to stress.

## Conclusions

In this study, 19 ZjSWEET family members of jujube were identified and were found to be distributed on eight jujube chromosomes. The ZjSWEETs were divided into 4 groups by phylogenetic analysis. The similar homologous genes in the topology have similarly conserved motifs and gene structures. *Cis*-acting elements related to hormones, stress, and growth were identified in the upstream sequence of the ZjSWEETs promoter. The expression of ZjSWEETs is tissue-specific and specific to the developmental stage. *ZjSWEET11* and *ZjSWEET18* gradually increased with the development of the fruit and reached a high level at the full-red fruit stage. ZjSWEETs are involved in the response of jujube to abiotic stresses including low temperature, salt, and alkaline conditions. *ZjSWEET2* was highly induced in response to cold stress, and *ZjSWEET8* was significantly up-regulated in response to alkali and salt stresses. This study provides a reference basis for further exploring the function of ZjSWEETs and analyzing their regulatory role in fruit sugar accumulation and abiotic stress responses in jujube.

##  Supplemental Information

10.7717/peerj.14704/supp-1 Supplemental Information 1 The protein sequences of SWEET genes from *Ziziphus jujuba* Mill

10.7717/peerj.14704/supp-2 Supplemental Information 2 The CDS sequences of ZjSWEETs

10.7717/peerj.14704/supp-3 Supplemental Information 3 The motif logos of ZjSWEETs corresponding to the phylogenetic tree

10.7717/peerj.14704/supp-4 Supplemental Information 4 The protein sequences of AtSWEETs and MdSWEETs

10.7717/peerj.14704/supp-5 Supplemental Information 5 The sequence of 2 kb upstream of the ZjSWEET gene

10.7717/peerj.14704/supp-6 Supplemental Information 6 The primer sequences of ZjSWEET genes for qRT-PCR

10.7717/peerj.14704/supp-7 Supplemental Information 7 The raw data of Ct value used for qPCR in Figure5

10.7717/peerj.14704/supp-8 Supplemental Information 8 The raw data of Ct values used for qPCR in Figure6

10.7717/peerj.14704/supp-9 Supplemental Information 9 Agar gel electrophoresis of qPCR products of ZjSWEETs

## Additional Information and Declarations

### Data Availability

The following information was supplied regarding data availability: The raw data is available in the Supplementary Files.
