# Supplementary figures and images for: Genome-wide identification and expression profile analysis of SWEET genes in Chinese jujube
Source: PeerJ. 2023 Jan 17;11:e14704. doi: 10.7717/peerj.14704 (PMC9854374; doi:10.7717/peerj.14704)

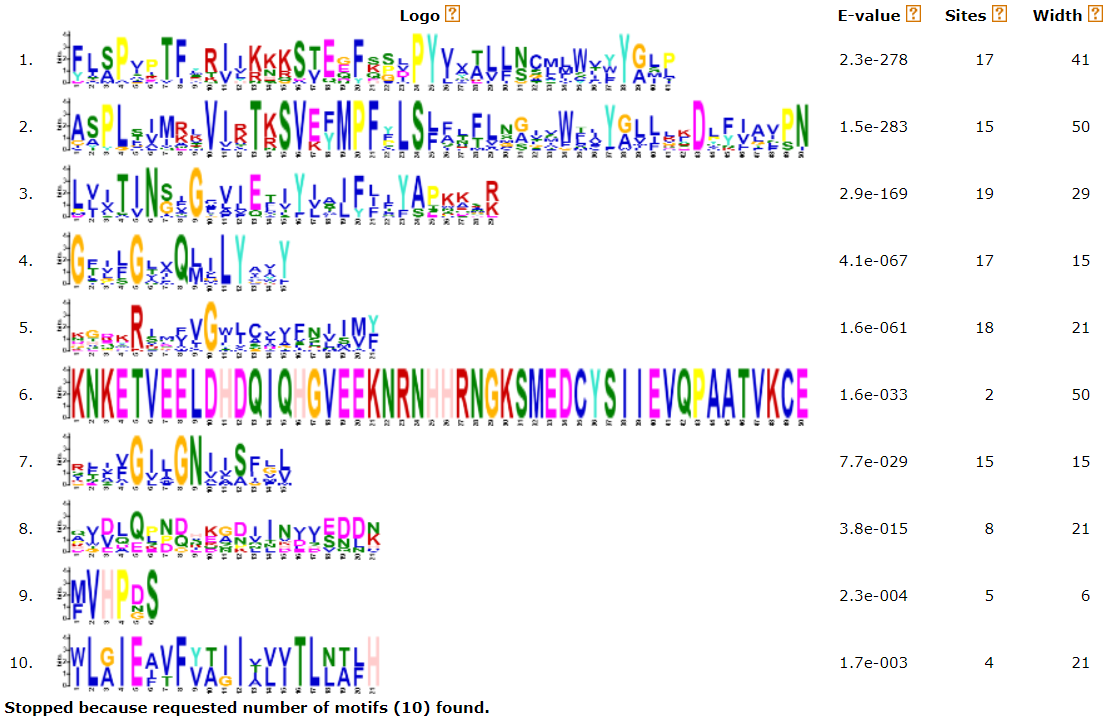

Supplement: Supplemental Information 3 [file peerj-11-14704-s003.png]

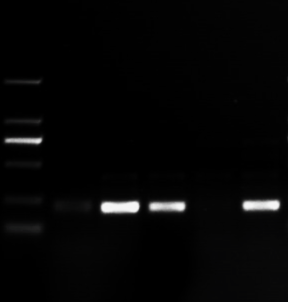

Supplement: Supplemental Information 9 [file peerj-11-14704-s009.zip › ZjSWEET18.png]

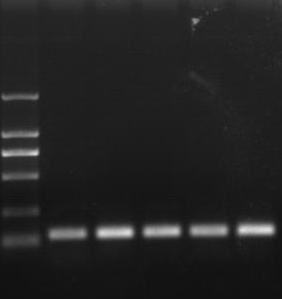

Supplement: Supplemental Information 9 [file peerj-11-14704-s009.zip › ZjACT.png]

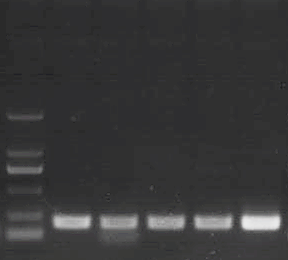

Supplement: Supplemental Information 9 [file peerj-11-14704-s009.zip › ZjSWEET1.png]

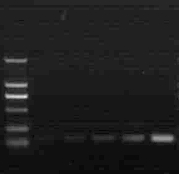

Supplement: Supplemental Information 9 [file peerj-11-14704-s009.zip › ZjSWEET2.png]

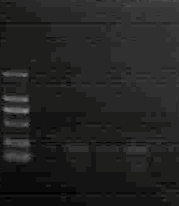

Supplement: Supplemental Information 9 [file peerj-11-14704-s009.zip › ZjSWEET3.png]

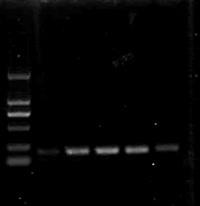

Supplement: Supplemental Information 9 [file peerj-11-14704-s009.zip › ZjSWEET4.png]

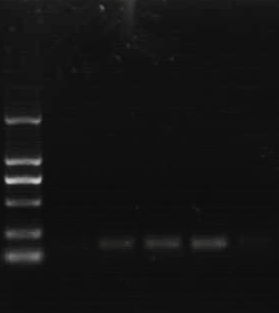

Supplement: Supplemental Information 9 [file peerj-11-14704-s009.zip › ZjSWEET6.png]

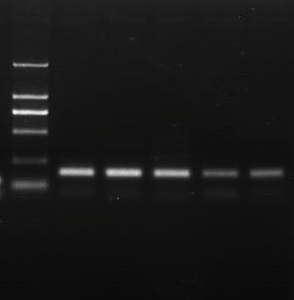

Supplement: Supplemental Information 9 [file peerj-11-14704-s009.zip › ZjSWEET8.png]

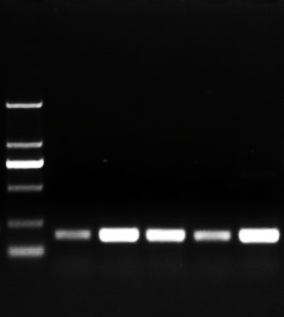

Supplement: Supplemental Information 9 [file peerj-11-14704-s009.zip › ZjSWEET11.png]

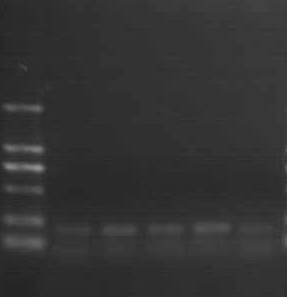

Supplement: Supplemental Information 9 [file peerj-11-14704-s009.zip › ZjSWEET12.png]

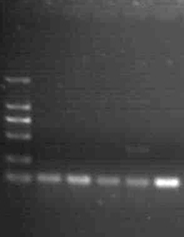

Supplement: Supplemental Information 9 [file peerj-11-14704-s009.zip › ZjSWEET13.png]

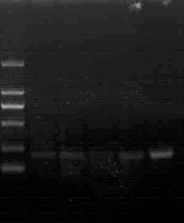

Supplement: Supplemental Information 9 [file peerj-11-14704-s009.zip › ZjSWEET15.png]
